# Structural insights into the targeting of mRNA GU-rich elements by the three RRMs of CELF1

**DOI:** 10.1093/nar/gkt470

**Published:** 2013-06-06

**Authors:** John M. Edwards, Jed Long, Cornelia H. de Moor, Jonas Emsley, Mark S. Searle

**Affiliations:** ^1^School of Chemistry, Centre for Biomolecular Sciences, University Park, University of Nottingham, Nottingham NG7 2RD, UK and ^2^School of Pharmacy, Centre for Biomolecular Sciences, University Park, University of Nottingham, Nottingham NG7 2RD, UK

## Abstract

The CUG-BP, Elav-like family (CELF) of RNA-binding proteins control gene expression at a number of different levels by regulating pre-mRNA splicing, deadenylation and mRNA stability. We present structural insights into the binding selectivity of CELF member 1 (CELF1) for GU-rich mRNA target sequences of the general form 5′-UGU*N*_x_UGU*N*_y_UGU and identify a high affinity interaction (K_d_ ∼ 100 nM for x = 2 and y = 4) with simultaneous binding of all three RNA recognition motifs within a single 15-nt binding element. RNA substrates spin-labelled at either the 3′ or 5′ terminus result in differential nuclear magnetic resonance paramagnetic relaxation enhancement effects, which are consistent with a non-sequential 2-1-3 arrangement of the three RNA recognition motifs on UGU sites in a 5′ to 3′ orientation along the RNA target. We further demonstrate that CELF1 binds to dispersed single-stranded UGU sites at the base of an RNA hairpin providing a structural rationale for recognition of CUG expansion repeats and splice site junctions in the regulation of alternative splicing.

## INTRODUCTION

The CELF family of RNA-binding proteins, which includes CELF1 (CUG-BP1) in humans, ETR-3-like factors, embryonic deadenylation element binding protein (EDEN-BP) in *Xenopus* and the highly homologous Bruno in *Drosophila* (1–5), are widely conserved in nature and regulate multiple facets of gene expression at the level of alternative splicing of mRNA, through translational regulation, deadenylation and mRNA stability (6–13). In mice, CELF1 upregulation leads to a phenotype similar to myotonic dystrophy (DM) (14,15) and has been implicated in muscle-specific disease, aging and cognitive impairment in humans (16–18). Homologous CELF1 proteins are ubiquitously expressed and involved in a variety of important physiological and pathological cellular processes linked to cell differentiation, proliferation and inflammation (2,13,19–26).

Investigations of CELF1 function in humans have largely focused on its role in regulating alternative splicing and ability to modulate translation of mRNAs. In *Xenopus*, CELF1 induces deadenylation and appears to play a key role in regulating poly(A) shortening (27–29). Recent studies, using *in vitro* assays for assessing mRNA decay in AU-rich mRNAs, have shown that CELF1 binds specifically to mRNAs and stimulates poly(A) shortening in the 3′ untranslated region (3′ UTR) by recruiting the poly(A) deadenylase ribonuclease (PARN) (13,30). In this case, binding to target EDEN mRNA GU-rich elements (GREs) is necessary for function (2,12,31–35), with EDEN-dependent deadenylation regulated by neighbouring *cis*-acting AU-rich elements (AREs) (13). In mammalian cells, ARE-binding proteins have been identified that both positively and negatively regulate mRNA decay or translation through interactions with the deadenylation machinery. CELF1 and CELF2 have overlapping but non-identical roles in the regulation of alternative splicing and can promote both exon skipping and exon inclusion (18). CELF2 has been best studied in splicing, and its binding in the vicinity of the branchpoint has been reported to promote skipping of the downstream exon, whereas binding downstream of an exon can promote its inclusion (36,37). These long-range interactions have been reported to be mediated through an interaction between CELF2 and the U2 snRNP, but this interaction appears to be absent for CELF1 (38).

The CELF proteins consist of three 80–100 residue RNA recognition motifs (RRMs) all of which adopt the same structural topology with a four-stranded anti-parallel β-sheet packed against two α-helices (39,40). The RNA-binding surface lies across the face of the β-sheet with individual RRMs recognising 3–4 nt of single-stranded RNA via a number of conserved residues that facilitate π-stacking and hydrogen bonding contacts. Structural studies indicate that the third RRM of human CELF1 has an N-terminal extended region of 7 residues, which interacts with the β-sheet and extends the mRNA binding surface. The two N-terminal motifs (RRMs 1 and 2) are connected by a short 8-residue linker, which allows conformational flexibility and co-operative high affinity binding to tandem UGU sites (40,41). In contrast, the third domain in full-length CELF1 proteins is separated by a much longer unstructured linker sequence of variable length (160–230 residues).

Although the individual domains of CELF1 have been studied on multiple RNA substrates and early investigations identified binding to CUG repeats (32,42–45), GREs appear to be the most abundant targets for CELF proteins. The 15-mer RNA sequence 5′-UGUUUGUUUGUUUGU (EDEN15) represents the consensus EDEN-binding motif identified by systematic evolution of ligands by exponential enrichment (SELEX) methods (12,46). Although the GRE has a high density of UGU sites, there is some apparent redundancy in accommodating multiple RRMs in close proximity (40,41,45). However, many well-characterized natural binding sequences consist of more dispersed UGU repeats (7,13,21,31,46,47) that may be able to engage with all three domains of CELF1 proteins simultaneously.

A number of mammalian mRNAs containing multiple UGU sites for CELF1 have recently been described (13), with the *c*-fos and TNFα RNA substrates within AREs shown to enhance mRNA decay in HeLa extracts. In these studies, a synthetic GU-rich EDEN motif of 19 nt (EDEN19) derived from the 3′ UTR of *Xenopus c*-mos was shown to be a competitive inhibitor in *in vitro* deadenylation assays (Table 1). The same study reported *in vitro* assays which show that CELF1 enhances deadenylation of these RNA substrates. The observation that the poly(A) deadenylase PARN is co-immunodepleted with CELF1 strongly suggests that PARN and CELF1 form a protein-mediated complex (30). Glutathione S-transferase (GST) pull-down assays and detection by western blotting confirmed that the two proteins have a direct co-interaction, and this may be regulating deadenylation rates, rather than binding to a common RNA substrate, increasing processivity and poly(A) shortening.

**Table 1.** Binding sequences for CELF1 from *c*-fos, *c*-jun, TNFα and *c*-mos (13), and consensus EDEN sequences for binding of CELF1 *Xenopus* to a single RNA substrate *via* multiple RRMs (UGU motifs underlined)

Native CELF1 target sequences	
*c*-fos:	UGUUCAUUGUAAUGUU
TNFα:	UGUUCCCAUGU … N_>40_ … UGU
*c*-jun:^a^	UGUU*UGGG*UAUCCUG*CCCA*GUGUUGUUUGU …
*c*-mos:	UAUAUGUAUGUGUUGUUUUAUGUGUGUGUGUGUGCU
EDEN19:	UGUAUGUGUUGUUUUAUGU
Designed sequences	
GRE (EDEN11):	UGUUUGUUUGU
EDEN15:	UGUUUGUUUGUUUGU
EDEN-2U/4U:	UGU*UU*UGU*UUUU*UGU
EDEN-2U/1U:	UGU*UU*UGU*U*UGU
EDEN-4U/2U:	UGU*UUUU*UGU*UU*UGU
EDEN-4U:	UGUUUUUUGU
EDEN7:	UGUUUGU
EDEN-2U/HL:^a^	UGU*UU*UGU*UCCCGAGGACGGGU*UGU

^a^Sequences shown in italics for *c*-jun and EDEN-2U/HL are able to form a hairpin stem-loop.

The structural basis for the binding of the full-length CELF1 to its target RNA substrate is still not fully understood. It is unclear whether all three domains of the CELF1 protein are simultaneously recruited to a single RNA-binding element, what the sequence requirements are for this interaction and whether there is selectivity in the arrangement of the three RMMs in this recognition process. Using a stable three-domain CELF1 construct (RRM123), we describe nuclear magnetic resonance (NMR) structural and biophysical studies based on chemical shift perturbation (CSP) analysis, paramagnetic relaxation enhancement (PRE) experiments with spin-labelled RNA substrates and binding affinity studies by isothermal titration calorimetry (ITC). A family of target RNA sequence of the form 5′-UGU*N*_x_UGU*N*_y_UGU, with three variably spaced RRM UGU-binding sites, has enabled us to identify a high affinity RNA motif (K_d_ ∼ 100 nM) that permits simultaneous interaction of all three RRMs within a single 15-nt substrate. The PRE experiments demonstrate selective signal attenuation in NMR spectra that are consistent with binding selectivity and a non-sequential arrangement of RRMs on consecutive UGU sites along the RNA substrate.

CELF1 is causally associated with DM type 1 where the toxic effects of the RNA 3′-UTR (CUG)_n_ expansion in the mutant RNA is mediated primarily by the RNA-binding protein MBNL1 (muscleblind-like 1) (48–51). The binding of CELF1 to double-stranded CUG repeats was shown to be localized to the single-stranded regions at the base of the RNA hairpin by electron microscopy (52), but the underlying basis of this interaction at the level of the individual RRMs has not been described. We present a structural rationale for the interaction of CELF1 with dispersed UGU sites, across the base of a double-stranded hairpin loop structure which facilitates remodelling of RNA conformation as well as regulating alternative splicing by binding to UGU sites in intronic regions adjacent to and across splice site junctions.

## MATERIALS AND METHODS

### Protein expression and purification

A construct of full-length *Xenopus* CELF1 was cloned into the pET28a vector. The RRM123 construct was produced by deletion of the codons for residues 215–384 using one-step PCR. The RRM23 construct was produced by the further deletion of the codons for residues 1–107 using the same method. All protein constructs were expressed in *Escherichia coli* BL21 (DE3) cells as fusions with an N-terminal histidine tag. Growths were carried out in Luria broth for unlabelled material and M9 minimal media for production of isotopically labelled protein. In all, 30 µg/ml kanamycin was used as an antibiotic control in all growths. Protein expression was induced with 1 mM isopropyl β-D-1-thiogalactopyranoside (IPTG) for 16 h at 30°C.

Cells were harvested by centrifugation and lysed by sonication. The lysis buffer for RRM123 was 25 mM potassium phosphate, 500 mM NaCl (pH 7.5). For wild-type CELF1, the lysis buffer was 25 mM potassium phosphate, 50 mM NaCl and 4 M urea. In all cases, a protease inhibitor cocktail (Roche) was added to the lysis buffer immediately before sonication. Cell debris was removed by centrifugation of the lysate, which was then applied to a His-Pur cobalt affinity column and left for 2 h at 4°C.

This column was then washed with 25 ml of a high salt buffer [2 M NaCl, 25 mM potassium phosphate (pH 7.0)] followed by a 10 mM imidazole wash to remove non-specifically binding proteins. RRM123 was eluted into 25 mM potassium phosphate, 200 mM NaCl and 500 mM imidazole (pH 7.0). Wild-type CELF1 was eluted in 25 mM potassium phosphate, 50 mM NaCl, 2 M urea and 500 mM imidazole. RRM123 was purified by gel filtration using a Superdex 200 column and desalted into water. Wild-type CELF1 was immediately desalted into water using a HiTrap desalting column (Amersham Biosciences). The eluted proteins were immediately frozen using liquid nitrogen and then lyophilised and stored at −20°C.

### NMR spectroscopy

NMR samples of RRM123 were prepared by dissolving lyophilised protein in 25 mM potassium phosphate, 100 mM NaCl, 10% (v/v) D_2_O (pH 7.0). The RRM123 construct contains seven Cys residues, which could potentially lead to aggregation through disulfide bond formation. NMR sample stability studies showed little evidence of significant degradation over NMR acquisition periods of >96 h even in the absence of DDT. Wild-type CELF1 required the addition of 200 mM urea to the NMR buffer for solubilization of the protein. Experiments were conducted at 25°C on an 800 MHz Bruker Avance III Spectrometer with cryoprobe. Protein concentrations of 200–250 µM were used for all RRM123 experiments. Wild-type CELF1 experiments used protein concentrations of ∼100 µM. Data were collected using Topspin 2.3 and 3.0 and processed in CCPN Analysis version 2.1.

All RNA molecules were supplied by Dharmacon. Spin-labelled RNA for PRE studies was produced by incorporating 4-thiouridine at a specific site with subsequent derivatization of the nucleobase to form a disulfide using three equivalents of (1-oxyl-2,2,5,5-tetramethylpyrroline-3-methyl)methanethiosulphonate (MTSL) for 2 h in darkness at room temperature, following the procedure of Qin *et al.* (53). Unreacted MTSL was removed by loading the reaction mixture onto a HiTrap desalting column and eluting into RNAse free water. Spin-labelled RNA samples were used in the same buffered solutions described earlier in the text. The spin label was reduced at the end of the titration by addition of 5 equivalents of sodium ascorbate, after which the sample was left to react for 16 h at room temperature.

### ITC

ITC was carried out using a high sensitivity microcalorimeter driven by VPViewer2000 software (version 1.4.27) (MicroCal Inc, GE Healthcare). All buffered solutions were degassed for 10 min in a Thermovac (MicroCal) before loading. The cell was filled with 1.4 ml of 12.5 µM RRM123 in 25 mM potassium phosphate, 100 mM NaCl (pH 7.0) in RNase free water. In all, 300 µl of 125 µM RNA in the same buffer solution was loaded into the syringe. All titrations were carried out at 25°C. After an equilibration period of 20 min, 30 injections of 10 μl were added at 600 s intervals. A constant stirring speed of 300 rpm was applied throughout the titration to rapidly mix the contents of the cell. A reference power of 5 μcals^−^^1^ was used. Fitting of the resulting data was carried out using the Origin 7.0 fitting program using various binding models to determine ΔH, ΔS, K_d_ and the stoichiometry for the interaction. Baseline correction was necessary for some data sets.

### Modelling of RRM123 complexes using MD simulations

Models of the RRM123 construct bound to the EDEN-2U/4U and EDEN-2U/HL RNA sequences were constructed using the programme xLEAP within AMBER6 (54) by splicing together available crystal and NMR structures of the individual RRM complexes with RNA in the Protein Database (accession codes 3NMR, 3NNH and for RRM3, 2RQ4 and 2RQC) (39,40)*.* All hydrogen atoms were introduced, and suitable linkers constructed between the RRMs where these were not present in the available PDB files. TIP3P water was used as an explicit solvent and was added in a truncated octahedron geometry to a distance of 9 Å around the protein. Monovalent (Na^+^) counterions were added to ensure overall charge neutrality of the RNA phosphate backbone. Energy minimization was carried out using the SANDER modules of the AMBER program. Molecular dynamics simulations were run without restraints for up to 2 ns at 300 K. The resulting models were viewed using Pymol (55). The flexible N-terminus of the protein (residues 1–13) is absent in the available structures and is also omitted from the model. No evidence from NMR suggested that these played any role in RNA binding. The alignment of the RRMs on the RNA substrate was based on the PRE data where the domains were arranged in the order 2-1-3 from 5′ to 3′ on the RNA. A model of the 3-2-1 arrangement was also constructed and did not show any obvious steric problems.

## RESULTS

### Protein and RNA sample preparation

**Figure 1.**
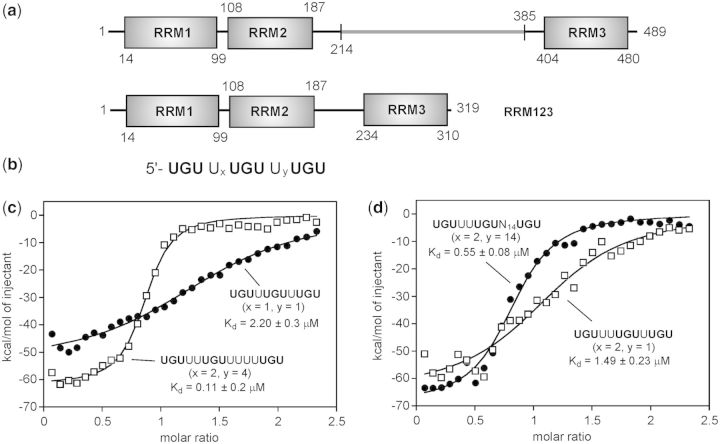
Full-length sequence of CELF1 showing RRM domains and linker sequences; alignment with the Δ(215–384) mutant RRM123 (**a**). RNA-binding sequence for CELF1 with three UGU sites separated by U_x_ and U_y_ spacers (**b**). Binding curves from ITC studies of CELF1 construct RRM123 with the RNA sequences EDEN11 (x = 1, y = 1) (GRE), EDEN-2U/4U (x = 2, y = 4) (**c**), and for EDEN-2U/1U (x = 2, y = 1) and EDEN-2U/HL (x = 2, y = 14) (**d**) (see sequences in Table 1). Total heat released is plotted as a function of the molar ratio of RNA to protein, and the non-linear least squares fit to the experimental data are shown as a solid line. Dissociation constants, binding stoichiometries and enthalpies of binding are shown in Table 2.

His-tagged bacterial expression constructs were produced for the individual CELF1 RRMs 1, 2 and 3, for a fragment containing domains 1 and 2 (RRM12) and for the full-length 489 residue CELF1 protein using methodology previously described (33). The isolation of full-length CELF1 was particularly problematic with the protein readily degrading through proteolysis of the linker region. To address this a three-domain construct RRM123 with the linker sequence truncated to 35 residues Δ(215–384) (Figure 1a) proved to be more stable, water soluble and amenable to biophysical investigation.

On the basis of previously reported potential CELF1-binding sites identified in *c*-fos, TNFα, *c*-jun and *c*-mos (Table 1), which contain either multiple UGU sites in close proximity, tracts of GU repeats and/or more widely dispersed UGU sites (13), the consensus sequence UGU*N*_x_UGU*N*_y_UGU (Figure 1a) was used to investigate the simultaneous binding of all three RRMs to a single RNA element and examine the UGU spacing requirements for this interaction. The analysis of more dispersed UGU sites is more challenging because formation of RNA secondary structure is potentially influential in bringing distant UGU sites into close proximity and may account for CELF1 binding to, and deadenylation of, some known natural mRNAs. The *c*-jun ARE (Table 1) is predicted to form a stable hairpin with a stem-loop inserted between potential UGU binding sites (bases shown underlined).

### Binding affinities for multiple RRM interactions

The affinity and binding stoichiometry of RRM123 with a number of RNA substrates of the general sequence 5′-UGU*N*_x_UGU*N*_y_UGU was examined by ITC in buffered solutions containing 25 mM potassium phosphate and 100 mM NaCl at pH 7.0 (Figure 1). In all cases aliquots of a 125 µM solution of buffered RNA were titrated into a 12.5 µM buffered solution of protein. The consensus GRE (EDEN11; x = 1, y = 1) produced a binding isotherm with a K_d_ = 2.2 ± 0.3 µM and a binding stoichiometry *n* = 1.45 (Table 2). As a control, we repeated the experiment with EDEN-4U (UGUUUUUUGU) in which deletion of the central guanine eliminates the possibility of three UGU binding sites. Both the binding affinity and stoichiometry (K_d_ = 2.08 ± 0.4 µM, *n* = 1.64) are closely similar to EDEN11. In both cases, a binding stoichiometry mid-way between 1:1 and 1:2 (protein:RNA) is consistent with each RNA substrate carrying only two viable binding sites to accommodate the three binding motifs of RRM123.

**Table 2.** ITC analysis of RRM123 affinities and binding stoichiometriesfor various RNA substrates

RNA	K_d_ (µM)	N (sites)	ΔH (kcal/mol)
UGUUUGUUUGU (EDEN11-GRE)	2.20 ± 0.3	1.45	−53.7 ± 1.4
UGUUUUUUGU (EDEN-4U)	2.08 ± 0.4	1.64	−54.4 ± 1.7
UGUAUGUGUUGUUUUAUGU (EDEN19)	0.33 ± 0.07	0.79	−111.4 ± 3.1
UGU*UU*UGU*UUUU*UGU (EDEN-2U/4U)	0.11 ± 0.02	0.85	−51.8 ± 1.0
UGU*UU*UGU*U*UGU (EDEN-2U/1U)	1.49 ± 0.23	1.19	−64.7 ± 2.9
UGU*UUUU*UGU*UU*UGU (EDEN-4U/2U)	0.98 ± 0.12	0.67	−35.7 ± 1.0
UGU*UU*UGU*UCCCGAGGACGGGU*UGU (EDEN-2U/HL)	0.55 ± 0.08	0.83	−69.0 ± 1.6

All data were collected at 25°C on 12.5 µM solutions of RRM123 dissolved in RNase free water buffered with 25 mM potassium phosphate and 100 mM NaCl at pH 7.0 and titrated with 300 µl of 125 µM RNA in the same buffer. Binding isotherms were fitted with MicroCal Origin software.

We turned to the longer RNA substrates with greater separation between UGU sites. ITC analysis of EDEN19, derived from the natural *c*-mos sequence, produced a binding stoichiometry closer to 1:1 (*n* = 0.79) and a 7-fold enhanced binding affinity (K_d_ = 0.33 ± 0.07 µM) compared with the EDEN11 GRE and EDEN-4U (Table 2). Putative UGU-binding sites on EDEN19 are identified in Table 1 (corresponding to site separations with x = 2 and y = 4). There is some potential degeneracy in how the various UGU sites could be occupied, which we attempted to resolve using the sequence EDEN-2U/4U with the same UGU site separation found in EDEN19. EDEN-2U/4U produced a high affinity interaction and binding stoichiometry (K_d_ = 0.11 ± 0.02 µM, *n* = 0.85) consistent with the simultaneous interaction of all three RRM motifs on the same RNA substrate (Figure 1). Switching the spacer lengths with EDEN-4U/2U, as evident for *c*-fos (Table 1), showed the same binding characteristics with a similar stoichiometry, but a 9-fold lower affinity (K_d_ = 0.98 ± 0.12 µM, *n* = 0.67), suggesting some selectivity in how the three domains are arranged on the RNA target.

We examined in more detail the effects of spacer separation between the second and third UGU sites while maintaining the optimum 2 U spacer for the tandem interaction of the two N-terminal RRMs (41). The sequence EDEN-2U/1U, with a single nucleotide spacer analogous to the EDEN11 GRE, gives a binding affinity for RRM123 of 1.50 ± 0.28 µM (*n* = 1.19), which is 15-fold weaker than for EDEN-2U/4U (Table 2). This is again consistent with steric constraints in accommodating all three motifs in such close proximity. The optimum spacing of UGU sites found in EDEN-2U/4U is also apparent in model structures containing motifs such as double-stranded hairpin loops. The folded secondary structure effectively brings three UGU sites into close proximity at the base of the hairpin. We tested this hypothesis with EDEN-2U/HL (x = 2, y = 14) where a hairpin loop of 14 nt was inserted into the expanded spacer sequence to mimic putative features evident in *c*-jun. EDEN-2U/HL bound to RRM123 with a 5-fold lower affinity than EDEN-2U/4U (K_d_ = 0.55 ± 0.08 µM, *n* = 0.83). However, the 1:1 stoichiometry similarly suggests that all three RRMs are engaged in binding interactions with available UGU sites.

### NMR studies of the RRM123–RNA interaction

**Figure 2.**
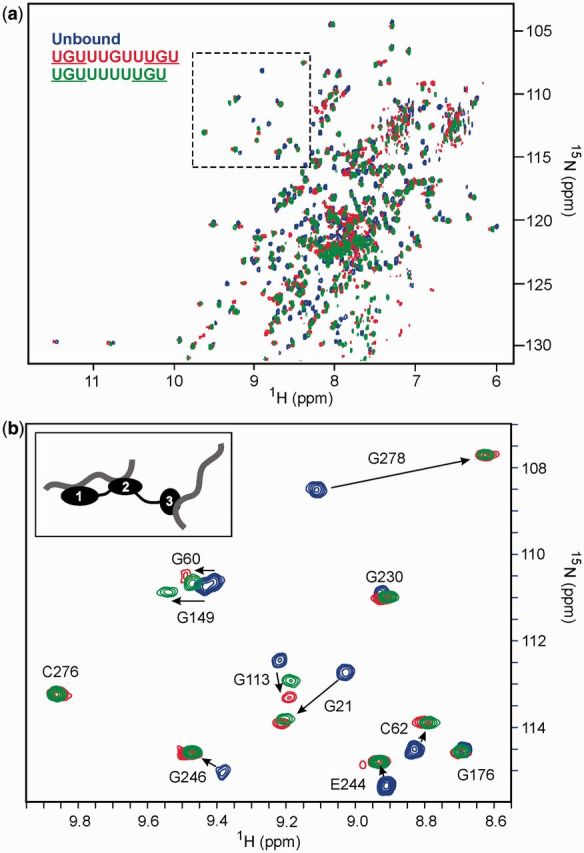
^1^H-^15^N TROSY spectra of the RRM123 construct at 800 MHz. Overlayed spectra of the unbound RRM123 (blue) with RRM123 complexes with EDEN11 (red) and EDEN-4U (green) at an RNA:protein ratio of 3:1. The region shown highlighted in (**a**) containing cross-peaks of some Gly and Cys residues is expanded in (**b**). Key residues from all three RRMs that show significant CSP effects on RNA binding are labelled. The proposed binding stoichiometry is shown schematically.

We examined the mode of interaction of ^15^N-labelled RRM123 with a number of RNA substrates by NMR at 800 MHz at protein concentrations of ∼200–250 µM [25 mM potassium phosphate, 100 mM NaCl (pH 7.0)]. The ^1^H-^15^N transverse relaxation optimized spectroscopy (TROSY) spectrum of the 319 residue RRM123 is well dispersed at 298 K with narrow line widths indicative of favourable internal dynamics between RRMs (Figure 2a). The spectrum of RRM123 is well represented by the sum of the three individual RRMs (CSP effects typically <0.05 ppm when comparing the three folded domains), consistent with the absence of significant interdomain interactions within RRM123. The remaining 35 residues within the RRM2-RRM3 linker sequence are located in a narrow overlapped region of the spectrum where the chemical shift dispersion is consistent with disordered structure. The ^1^H-^15^N heteronuclear nuclear overhauser enhancement (NOE) experiments show positive NOEs (>0.8) for the structured domains and zero or negative NOEs for the linker residues indicative of considerable flexibility, although in many cases, sequence specific assignments in the disordered region were not achieved.

We conducted RNA titration experiments with ^15^N-labelled RRM123 to further corroborate observed binding stoichiometries identified by ITC. The 11-mer GRE consensus sequence (UGUUUGUUUGU) was used as a control, representing the minimum binding element with three possible UGU binding sites in close proximity. In ^1^H-^15^N-TROSY spectra, a 1:1 ratio of RNA:protein resulted in extensive line-broadening across all three RRMs and the loss of many resonances, suggesting conformational exchange on an intermediate to slow timescale. However, increasing concentrations up to a 2:1 ratio of RNA:protein produced considerable sharpening, with bound peaks visible for all three domains. Residues G21 and C61 (RRM1), G113 and C150 (RRM2) and G278 (RRM3) are particularly sensitive to RNA binding and are all seen to undergo significant CSPs in the bound state (Figure 2b). NMR titration with EDEN-4U, with only two UGU sites, produced CSP effects at binding saturation and narrow linewidths that were similar to those with EDEN11 (Figure 2b). Both the NMR and ITC data concur that the EDEN11 GRE accommodates RRMs at only two of the three UGU sites with binding saturation of RRM123 achieved by recruiting a second RNA substrate (see schematic inset in Figure 2b). In the context of a natural mRNA target, this could be an intramolecular interaction with a more distant UGU motif with the GRE providing the anchor point for the interaction.

**Figure 3.**
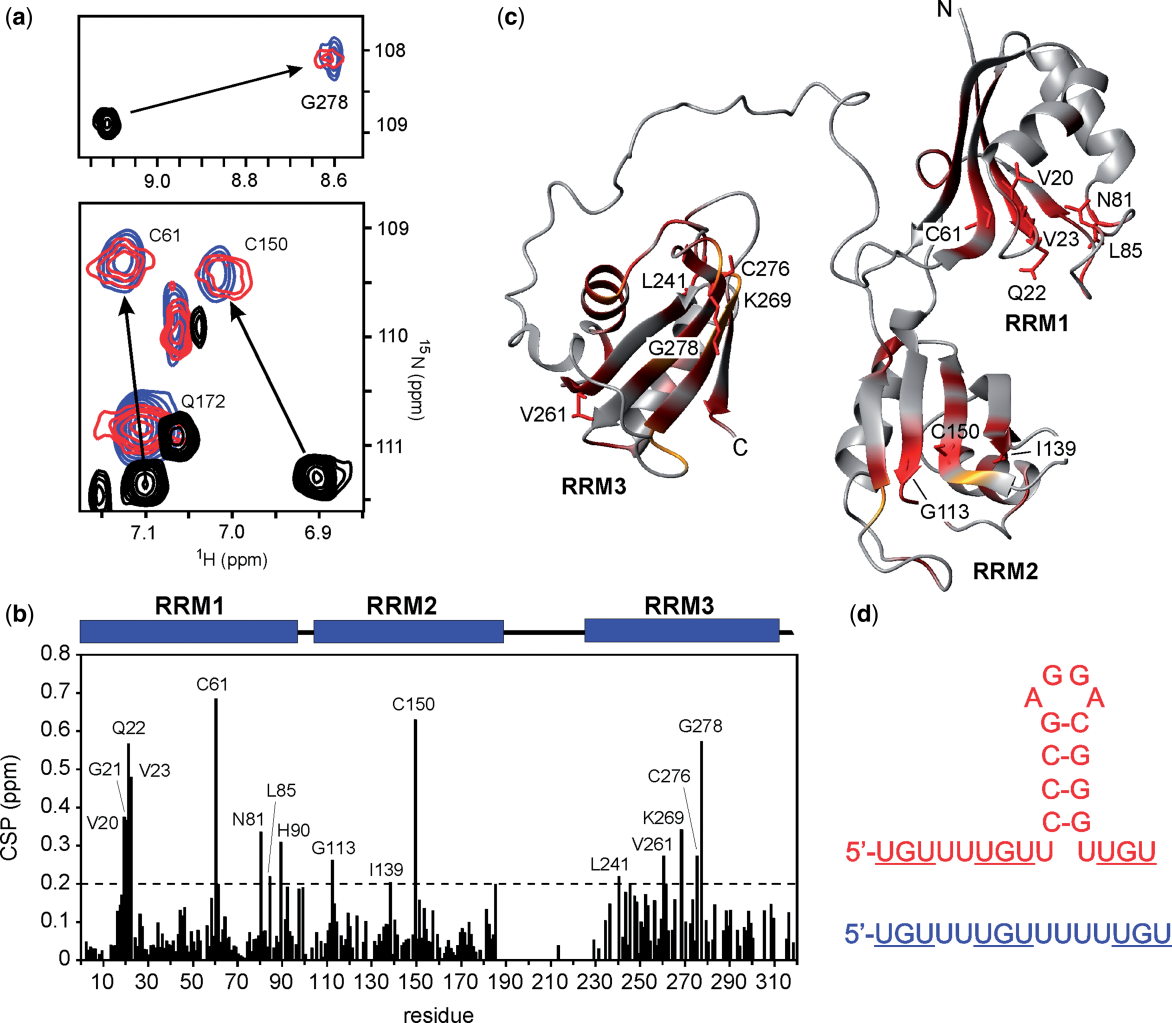
NMR analysis for binding of RRM123 to the high affinity sequences EDEN-2U/4U and EDEN-2U/HL. (**a**) Overlayed portions of the ^1^H-^15^N-TROSY spectrum at 800 MHz of RRM123 (unbound in black) showing large CSPs for C61 (RRM1), C150 (RRM2) and G278 (RRM3) at a 1:1 binding ratio with the RNA sequences EDEN-2U/4U (blue) and EDEN-2U/HL, (red); (**b**) histogram of CSPs for RRM123 complex formation with EDEN-2U/4U with CSP effects >0.2 ppm labelled; (**c**) model of the RRM123 structure showing all CSPs >0.1 in red with residue side chains illustrated for CSPs > 0.2 ppm. Some residues broaden and disappear completely and are coloured in yellow; (**d**) RNA sequences coloured to match spectra in (**a**), with UGU-binding sites underlined.

In contrast, the NMR titration of RRM123 with the high affinity EDEN-2U/4U RNA substrate resulted in slow-exchange binding across all three domains with binding saturation reached at close to a 1:1 ratio. Bound resonances showed significantly increased line widths consistent with slower tumbling of the complex. CSP effects were readily apparent across all three RRMs with C61 (RRM1), C150 (RRM2) and G278 (RRM3) particularly affected (Figure 3a–c), confirming the simultaneous engagement of all three domains. No further CSP changes were apparent at higher ratios of RNA:protein up to 3:1.

Titration experiments with EDEN-2U/HL (Figure 2d), in which UGU sites are located at the single stranded 5′ and 3′ tail ends of a 14 nt hairpin loop, showed similar effects. The ^1^H-^15^N-TROSY spectra of the complex showed even greater line broadening effects, which precluded an extensive CSP analysis. However, key resolved residues (C61, C150 and G278) within each of the three domains showed similar CSP effects to EDEN-2U/4U, as shown overlayed in Figure 3a. The ^1^H NMR spectra of RRM123 bound to EDEN-2U/HL revealed imino proton signals between 12.5 and 13.5 ppm that were consistent with four stable Watson–Crick base pairs within the hairpin double-stranded stem region. In both complexes, a combination of ITC and NMR data strongly support formation of 1:1 complexes in which all three RRMs simultaneously interact with the three UGU sites on a single RNA substrate.

### Paramagnetic relaxation spin labelling of the RNA substrate reveals binding selectivity

**Figure 4.**
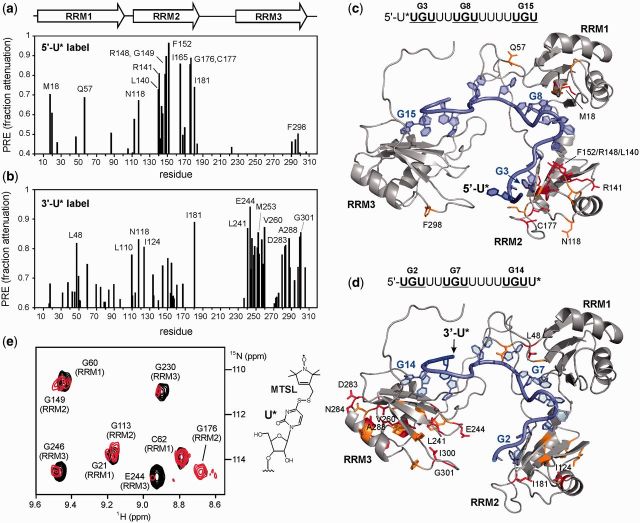
PRE effects in binding of RRM123 to spin-labelled EDEN-2U/4U substrates with MTSL covalently attached through 4-thiouridine (U*) at the 5′ or 3′ terminus. (**a**) Histogram showing PRE effects from 5′-U* across all three domains with the largest attenuation effects >0.7 clustered in RRM2. (**b**) PRE effects from 3′-U* with specific effects now evident in RRM3; (**c**) RNA sequence and model of the structure of 5′-U* labelled EDEN-2U/4U with RRM123 showing the location of the largest PRE effects. All residues with PREs >0.7 are shown in red. With the exception of Met18, all of these are in RRM2. Residues with PREs between 0.5 and 0.7 are shown in orange. The modified base to which the MTSL tag is attached (U*) is shown in blue. The RNA sequence used in the modeling is also shown with the three guanines highlighted on the structure; (**d**) PRE effects from 3′-U* labelled EDEN-2U/4U, with residues with PRE effects >0.8 shown in red (>0.7 in orange); (**e**) overlayed portions of the TROSY spectra of the complexes of RRM123 with 5′-labelled EDEN-2U/4U (black) and 3′-labelled EDEN-2U/4U (red) showing differentiation signal attenuation effects particularly for G176 (RRM2) and E244 (RRM3). The structure of MTSL modified 4-thiouridine (U*) is also shown.

A paramagnetic spin label (MTSL) was covalently attached to the RNA substrate through the incorporation of the nucleobase analogue 4-thiouridine (U*) (Figure 4). Relaxation enhancement effects in TROSY spectra were used to identify the relative positioning and alignment of the three RRMs of RRM123 bound to the high affinity EDEN-2U/4U RNA substrate (53,56). We first showed that a tetranucleotide RNA fragment 5′-U*UGU, with the MTSL at the 5′-terminus, was able to bind to the isolated RRM1 and produce specific signal attenuations through PRE effects across the β-sheet binding surface, demonstrating that PREs can be used to map binding interfaces in good agreement with ^1^H-^15^N CSP methods (57,58).

**Figure 5.**
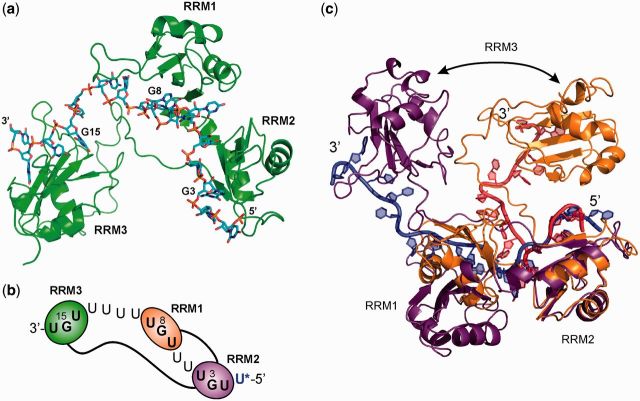
Structure and conformational flexibility of the RRM123 complex with EDEN-2U/4U. (**a**) Model refined from MD simulations showing the arrangement of the three RRMs in the order 2-1-3 from 5′ to 3′ along the RNA substrate. (**b**) The flexible 35-residue linker between RRM2 and RRM3 allows the C-terminal domain to fold back, as shown schematically. (**c**) Overlayed structures from the MD ensemble showing changes in orientation about the 8-residue RRM1-RRM2 linker sequence which allows RRM123 to adopt both compact and more extended conformations.

Subsequently, the high affinity RNA substrate EDEN-2U/4U was MTSL labelled through an additional U* at the 5′ terminus (Figure 4) and was isolated by gel filtration. We compared cross-peak intensities for RRM123 in ^1^H-^15^N TROSY spectra with those of the ascorbate-reduced RNA substrate with the spin label chemically quenched. The spin label produced a range of effects from either no attenuation to partial or complete signal broadening for a significant number of residues, suggesting a high degree of binding selectivity (Figure 4a). Mapping the PRE effects across the entire structure of RRM123 (Figure 4a and c) produced an attenuation pattern consistent with the strongest effects (>0.7) in RRM2. Smaller effects (<0.7, but more typically <0.5) were evident for RRM1 (Figure 4a), with the weakest perturbations (≤0.5) observed for RRM3. The PRE effects support a structural model in which RRM2 is primarily bound at the 5′-end of the labelled RNA substrate with RRM1 forming a tandem co-operative interaction at the adjacent UGU site (Figure 5). The flexible 35-residue RRM2-RRM3 linker allows RRM3 to fold back to complete the three-domain interaction by binding at the 3′-terminal UGU site.

We tested this model by incorporating the U* nucleobase analogue at the 3′ terminus of EDEN-2U/4U. Covalent modification with MTSL resulted in a contrasting pattern of signal attenuation in ^1^H-^15^N TROSY spectra (Figure 4b and d) with the strongest PRE effects (>0.7) now clearly evident for RRM3 where these were generally weak with the 5′-labelled RNA substrate (Figure 4a versus b). The overlay of the small region of the TROSY spectrum in Figure 4e shows differential effects, in particular for G176 (RRM2) and E244 (RRM3), in the presence of one or other spin-labelled RNA substrate that illustrate binding selectivity in the organization of the three RRMs in a non-sequential arrangement at UGU sites along the high affinity EDEN-2U/4U substrate.

### Modelling the structure and dynamics of the RRM123 interaction with RNA substrates

The trajectory of the RNA sequence across the binding surfaces of all three RRMs was modelled on the basis of NMR and X-ray structural data of complexes of the isolated domains (39,40), using the AMBER modelling suite with an explicit solvent model (see ‘Materials and Methods’ section). Initial models of the RRM123 complex with the high affinity EDEN-2U/4U substrate were arranged with the 2-1-3 alignment of RRMs from the 5′ to 3′ end of the RNA substrate, as supported by the PRE analysis. Various conformations of the flexible RNA spacer sequences between UGU sites were energy minimized and subjected to 1–2 ns of MD simulation to produce an ensemble of sterically and energetically plausible structures. The overall arrangement of the three motifs on EDEN-2U/4U is shown in Figure 5a and b. The conformational flexibility around the 8-residue RRM1–RRM2 linker results in different relative alignments of these two domains.

**Figure 6.**
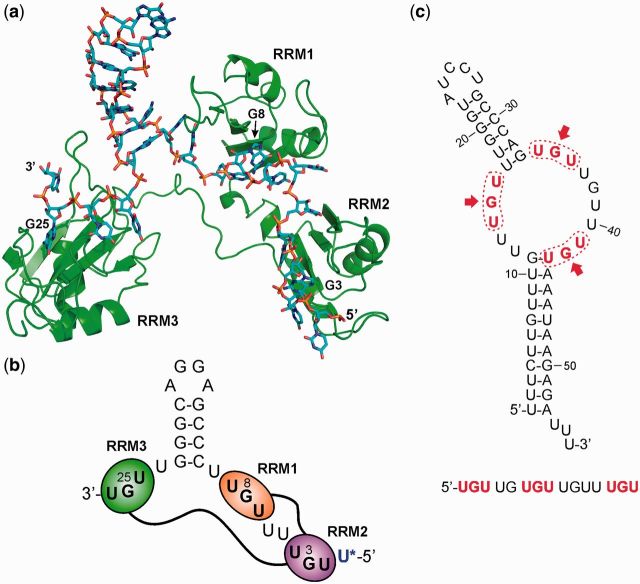
(**a**) Structural model from MD simulations of the complex of the CELF1 construct RRM123 with the hairpin-loop mRNA EDEN-2U/HL; (**b**) schematic representation for clarity. The RNA hairpin is effective in co-localizing all three single-stranded UGU-binding motifs, with RRM123 binding across the base of the hairpin stem-loop. (**c**) Mfold prediction of secondary structure formation in part of the 3′-UTR of *c*-jun, which triggers deadenylation. UGU-binding sites (highlighted in red) are founded within an unstructured GU-rich loop and are co-localized through the formation of two hairpin structures.

Structural studies of other tandem RRM partners of PABP, Sex-lethal (Sxl) HuD and Hrp1 RNA binding proteins bound to various different single-stranded substrates reveal RNAs with either a linear or kinked trajectory between RRM-binding surfaces (59–62). All of these are evident in the MD ensemble for the RRM123 complex with EDEN-2U/4U (Figure 5c) and suggest that intrinsic conformational flexibility is essential for optimizing the RNA-binding interface for targeting GREs through multiple interactions with variably spaced UGU sites. We also generated a number of energy minimized structures with the hairpin loop RNA substrate EDEN-2U/HL, one of which is shown in Figure 6a and b. The models show that the hairpin stem loop, containing four Watson–Crick hydrogen bonded base pairs, brings the three UGU sites within the 5′ and 3′ single-stranded tails into an alignment similar to that modelled with EDEN-2U/4U (Figure 5a). The RNA secondary structure and RRM interactions at UGU sites across the base of the hairpin motif remain stable throughout the 2 ns MD simulation.

## DISCUSSION

### Targeting of RNA substrates by CELF1 proteins

Recent studies have identified natural mammalian AREs containing GU-rich mRNA candidate-binding sites for CELF1 within HeLa cell extracts. The *c*-fos and TNFα AREs have been shown to enhance mRNA decay and contain multiple UGUA, UGUU and UGUG sequences in close proximity. The greater abundance of these sites than predicted from a random distribution suggests that these sequences contain high affinity CELF1-binding sites. A 19-nt fragment of *c*-mos (EDEN19) was shown to be a highly competitive inhibitor of deadenylation of *c*-fos and TNFα, suggesting that *c*-mos similarly contains one or more high affinity CELF1 recognition sites within the EDEN19 element (13). The analysis of CELF1-targeted 3′-UTRs of mRNAs in *Xenopus tropicalis*, using immunoprecipitated CELF1/mRNA complexes, identified an enrichment of putative EDEN sequences for which the EDEN11 motif (Table 1) with closely spaced UGU motifs represents the consensus-binding site (46).

We have studied a number of mRNA substrates of the general sequence 5′-UGUN_x_UGUN_y_UGU and have shown that a stable three-domain CELF1 construct binds with high affinity to the EDEN-2U/4U RNA motif (N = U, x = 2 and y = 4) derived from the *c*-mos natural target sequence. We have shown from ITC and NMR experiments, including PRE effects using spin-labelled RNAs, that the RRM123 CELF1 construct is able to simultaneously bind to the three UGU sites. The high affinity EDEN-2U/4U RNA substrate leads to binding selectivity in which the three RRMs of CELF1 are aligned in a non-sequential 2-1-3 arrangement from 5′ to 3′ along the RNA target sequence.

In contrast, our NMR and ITC studies of the consensus GRE identified by Vlasova *et al.* (12), which conforms to this general sequence but has single nucleotide spacers (x = 1 and y = 1) between UGU sites, is sterically unable to accommodate all three RRMs in such close proximity (40,41). We show that the GRE only partially binds the CELF1 protein through two of the three possible UGU-binding sites (5′-UGUUUGUUUGU). Thus, the GRE with sub-optimal spacing of UGU sites does not capture the full extent of CELF1-RNA recognition but may act as a key anchor point for the initial RNA interaction.

The recognition of UGU(U) motifs by the three RRMs is mediated by a common set of conserved residues on the surface of the β-sheet and adjacent loops that use a combination of base-specific hydrogen bonding and π-stacking interactions (39,40). Further selectivity for binding a GRE appears to be imparted by a *syn* glycosidic conformation for the guanine base, more typical of left-handed Z-RNA helices (40). RRM3 is, however, distinct in having an N-terminal extension that folds back to interact with the β-sheet and extend the RNA-binding surface (39). These differences may account for the 10-fold higher affinity of RRM3 for a UGU(U) motif (K_d_ ∼ 2–4 µM) compared with the other isolated RRMs (K_d_ ∼ 30–60 µM) (39–41).

However, RRM1 and RRM2 are separated by a short 8-residue linker and the tandem interaction of the two RRMs leads to high affinity co-operative binding (K_d_ ∼ 0.4–0.7 µM) to closely spaced UGU sites, realizing a >100-fold enhancement in affinity over the isolated domains (41). This tandem co-operative interaction is not evident for an RRM2-RRM3 construct (RRM23) with the 35 residue spacer where interactions between the two motifs are decoupled, resulting in a clear two-step binding process, which reflects the >10-fold difference in affinity between the two RRMs. This is evident from CSP effects with RRM23 in NMR titrations with a number of RNA substrates where perturbations were confined to RRM3 up to a 1:1 binding stoichiometry. ITC measurements indicated a first-step interaction with a K_d_ = 0.52 ± 0.12 µM for RRM3 binding to a UGU(U) site. We conclude that the co-operative binding of RRM1 and RRM2, which leads to a high affinity interaction, together with the strong binding of RRM3 in isolation, ensures that both ‘ends’ of CELF1 are anchored to an RNA substrate with comparable affinity, of particular relevance in targeting dispersed UGU-binding sites and in the structural remodelling of pre-mRNAs.

We tested this model with a designed RNA-binding element EDEN-2U/HL with UGU sites within the 5′ and 3′ single stranded tails at the base of a 14-nt hairpin loop. ITC and NMR titrations showed that RRM123 could bind with high affinity (K_d_ = 0.55 ± 0.08 µM) in a 1:1 binding event with NMR CSP effects consistent with all three motifs simultaneously engaging in RNA interactions. Bringing together distant UGU sites through RNA secondary structure formation may account for the observed interaction of CELF1 with mRNAs such as TNFα (13), which have a poor match to the consensus GRE. The known target *c*-jun was reported by Paillard *et al.* (47) to bind both *Xenopus* and human CELF1 and trigger rapid deadenylation when present in the 3′ UTR of mRNA. Vlasova *et al.* (12) reported a shorter version of the *c*-jun sequence, which could also trigger deadenylation. In both cases, the mRNA contains a consensus EDEN11 GRE site, but when considered as a linear sequence, it does not appear to contain a site capable of binding all three RRMs in the manner of a high affinity EDEN-2U/4U sequence. Secondary structure prediction for *c*-jun, using the Mfold web server (63), provides an explanation for binding of CELF1 to UGU motifs co-localized by the overall fold of the mRNA, as modelled in the complex with EDEN-2U/HL. The *c*-jun RNA shows two clear double-stranded regions (Figure 6c). One is from bases 17–31, which forms a hairpin held together primarily by Watson–Crick C-G base pairs, similar to the hairpin in the EDEN-2U/HL sequence. The second double stranded region is between bases 1–11 and 44–53, mostly composed of U-A base pairs. The two hairpins are linked by a single-stranded loop region, which contains five of the six possible UGU interaction sites in close proximity. The three highlighted UGU sites result in an arrangement analogous to that shown for EDEN-2U/HL, which is capable of binding all three RRMs of CELF1 with high affinity. There is also a reported dependence of deadenylation efficiency on the presence of AREs by Audic *et al.* (64), which are found in *c*-jun and TNFα. We can speculate that these elements facilitate formation of secondary structures that bring UGU sites into more favourable configurations for CELF1 binding, rather than having any direct interaction with CELF1.

This has possible implications both for defining the EDEN-binding motifs and for the role of CELF1 in regulating alternative splicing. Bioinformatic analysis by Masuda *et al.* (51) reported that partial CELF1 targets tend to cluster in intronic regions flanking alternative exons, with functional analysis consistent with CELF1 binding to the upstream intron facilitating exon skipping. Indeed, CELF1 can promote or inhibit exon inclusion depending on the location of recruitment to the transcript (65,66). It remains a possibility that CELF1 is able to bind across branch points or whole exons to regulate alternative splicing, with RRM1–RRM2 anchored on one side and RRM3 on the other, as illustrated for EDEN-2U/HL, providing a mechanism for exon exclusion.

Although we have worked largely with the truncated RRM123 construct in this study, for reasons of stability, ^15^N-labelled full-length *wt*-CELF1 (489 residues) with an additional 170 residues of flexible linker sequence was isolated in small amounts for RNA-binding studies with both EDEN-2U/4U and EDEN-2U/HL. Although the spectra of the *wt*-CELF1 in isolation were of high quality, owing to the intrinsic dynamics of the structure, RNA complex formation leads to extensive line broadening and poor resolution. Many of those residues that are expected to be perturbed (C61 in RRM1, C150 in RRM2 and G448 in RRM3), and a variety of residues usually associated with smaller CSPs show characteristic, although not readily quantifiable, binding effects that are consistent with all three RRMs of *wt*-CELF1 binding to these high affinity RNA substrates.

We also considered reports that differential phosphorylation of CELF1 has been implicated in regulating both protein–protein and protein–RNA interactions, with studies suggesting that phosphorylation of Ser28 in RRM1 of CELF1 increases RNA binding *in vitro* (67) and that hyperphosphorylation by protein kinase C (PKC) increases the half-life of CELF1 (68). To investigate the possible effects *in vitro*, we produced an S28D phosphomimetic mutant of the isolated RRM1. However, neither NMR nor ITC measurements could reveal any differences in binding affinity with GU-rich RNA substrates compared with the native RRM1 (data not shown).

The RNA mutation model for DM pathogenesis has shown that CUG expansion repeats in the 3′-UTR region lead to the formation of long double-stranded RNA hairpins that sequester RNA-binding proteins (48,49). The subcellular distribution of CELF1 and MBNL1, another developmentally regulated RNA-binding protein that controls alternative splicing, are of particular interest in this context. Although MBNL1 binds to double-stranded hairpins (50) and accumulates in nuclear foci (51), studies suggest that CELF1 is localized to the base of these RNA hairpin structures rather than the stem region (52). Our structural models, shown in Figure 5b, are consistent with this hypothesis and enable us to visualize how this might occur either through binding to an isolated single-stranded CUG repeat or to the single-stranded ‘tails’ at an RNA junction.

The RRM is a ubiquitous RNA-binding motif that is used in numerous post-transcriptional steps in gene expression and as a regulatory repressor of alternative splicing events. The polypyrimidine tract-binding protein (PTB) is a typical well-characterized example with a similar architecture to CELF1 of multiple flexibly linked RRMs, which bind pyrimidine-rich motifs (e.g. UCUU, CUCUCU) (69–71) within regulatory elements of RNA (72–75). PTB suppresses splicing by blocking various components of the spliceosome, including the splicing factor U2AF65 (76,77), or by masking splice sites through formation of oligomeric assemblies across intron–exon boundaries (78). Alternative indirect mechanisms suggest that PTB blocks splicing complex formation through its ability to remodel pre-mRNA by bringing together distal regions to form looped structures or by formation of non-productive interactions with other stem-loop motifs (79–83). Although full-length PTB has eluded structure determination, the structure, affinity and binding specificity of the individual RRMs have been reported.

It is also apparent that PTB mediates both RNA and protein interactions. The PTB–Raver1 interaction, which modulates splicing of α-tropomyosin (Tpm1), is well characterized structurally and functionally (84,85). Structures derived from Raver1 peptide-RRM complexes reveal the versatility of RRMs as a recognition motif in mediating protein interactions across the distal helical face while engaged in RNA binding across the β-sheet. By analogy, recent studies using *in vitro* assays for assessing mRNA decay in AU-rich mRNAs have shown that CELF1 binds specifically to mRNAs and stimulates poly(A) shortening in the 3′ UTR by recruiting the poly(A) deadenylase PARN (13,30). Although the details of this interaction are still ill-defined, it would appear that a Ser-rich motif within the RRM2-RRM3 flexible linker region of CELF1, close to a putative amphipathic helical motif (residues 332–340), facilitates recruitment. Phosphorylation-regulated CELF1 recruitment of PARN to initiate deadenylation and mRNA decay has been proposed (86) and is consistent with functional studies of CELF1 (4) in which a G331D mutation appears to knock-out CELF1 as a deadenylation factor. As a step towards elucidating protein–protein and protein–mRNA interactions of CELF1 linked to mRNA functional regulation, we have presented new structural insights into the selectivity and affinity of the three RRMs of CELF1 for a GU-rich mRNA target sequence containing multiple UGU-binding sites.

## FUNDING

The authors acknowledge funding from the Biotechnology and Biological Sciences Research Council (BBSRC) to M.S.S. and J.L. (BB/I011420/1) and studentship funding (to J.E.) from a BBSRC Doctoral Training Grant and from the School of Chemistry, University of Nottingham. The NMR facilities were supported by the University of Nottingham through the Capital Infrastructure Fund. Funding for open access charge: University of Nottingham.

*Conflict of interest statement*. None declared.
